# Sperm morphology and count vary with fine-scale changes in local density in a wild lizard population

**DOI:** 10.1007/s00442-019-04511-z

**Published:** 2019-10-17

**Authors:** Matthew C. Kustra, Ariel F. Kahrl, Aaron M. Reedy, Daniel A. Warner, Robert M. Cox

**Affiliations:** 1 Department of Biology, University of Virginia, Charlottesville, VA 22904 USA; 2 Department of Ecology and Evolutionary Biology, University of California, Santa Cruz, CA 95064 USA; 3 Stockholm University, Zoologiska institutionen: Etologi, 106 91 Stockholm, Sweden; 4 Department of Biological Sciences, Auburn University, Auburn, AL 36849 USA

**Keywords:** Sperm competition, Postcopulatory sexual selection, Operational sex ratio, *Anolis sagrei*

## Abstract

Given that sperm production can be costly, theory predicts that males should optimally adjust the quantity and/or quality of their sperm in response to their social environment to maximize their paternity success. Although experiments demonstrate that males can alter their ejaculates in response to manipulations of the social environment and studies show that ejaculate traits covary with social environment across populations, it is unknown whether individual variation in sperm traits corresponds to natural variation found within wild populations. Using an island population of brown anole lizards (*Anolis sagrei*), we tested the prediction that sperm traits (sperm count, sperm morphology, sperm velocity) respond to natural variation in the risk of sperm competition, as inferred from the local density and operational sex ratio (OSR) of conspecifics. We found that males living in high-density areas of the island produced relatively larger sperm midpieces, smaller sperm heads, and lower sperm counts. Sperm traits were unrelated to OSR after accounting for the covariance between OSR and density. Our findings broaden the implications of sperm competition theory to intrapopulation social environment variation by showing that sperm count and sperm morphology vary with fine-scale differences in density within a single wild population.

**Electronic supplementary material:**

The online version of this article (10.1007/s00442-019-04511-z) contains supplementary material, which is available to authorized users.

## Introduction

Population density and sex ratio affect the probability of encountering both competitors and mates (McLain 1992; Kokko and Rankin 2006; McCullough et al. 2018). The frequency of these encounters can influence the intensity of male–male combat, the likelihood of mate acquisition, and the importance of sperm competition (Kokko and Rankin 2006; Knell 2009), potentially resulting in density-dependent mating tactics (Mobley and Jones 2007). For example, males may alter their allocation of resources to weapons or ornaments as a function of the density or sex ratio of their social environment (Gage 1995; Harris and Moore 2004; Buzatto et al. 2015). Although many studies have focused on the effects of density and sex ratio on precopulatory sexual selection (i.e., selection arising from variance in mating success), these aspects of the social environment can also influence postcopulatory sexual selection. Moreover, recent evidence suggests that the relative importance of postcopulatory processes may increase with population density (McCullough et al. 2018).

Sperm production can be energetically costly (Dewsbury 1982; Olsson et al. 1997; Kahrl and Cox 2015), and theory predicts that, all else being equal, males should invest more in ejaculate production as the risk of sperm competition increases (Parker 1993; Wedell et al. 2002; Parker and Pizzari 2010). Because the density and operational sex ratio (OSR, the ratio of sexually active males to sexually receptive females) of a population can each influence the risk of sperm competition (Lüpold et al. 2017), males may use these aspects of the social environment as cues to adaptively alter the ejaculate. Experimental data from laboratory settings show that sperm morphology and sperm count can change in response to manipulations of the social environment that alter the perceived level of competition for mates (Harris and Moore 2004; Crean and Marshall 2008; Ramm and Stockley 2009; Immler et al. 2010; Kelly and Jennions 2011; Firman et al. 2013; Moatt et al. 2014; Giannakara et al. 2016). Additionally, comparisons across populations have found associations between the risk of sperm competition and variation in ejaculate traits (Dziminski et al. 2010; Álvarez et al. 2013). Although these and other studies provide evidence for the plasticity of sperm phenotypes in response to manipulations of the social environment, it is unclear whether fine-scale variation in the social environment within natural populations may elicit a similar response.

In this study, we test the hypothesis that fine-scale variation in the social environment, as gauged by the local density of adult conspecifics and the local OSR, is associated with individual variation in sperm traits (sperm morphology, sperm count, and sperm velocity) across a wild population of brown anole lizards (*Anolis sagrei*). Male brown anoles likely experience strong postcopulatory sexual selection because females can store sperm for several months after mating (Calsbeek et al. 2007; Kahrl and Cox 2015), and typically produce offspring sired by several males (Calsbeek and Bonneaud 2008; Kamath and Losos 2018). Moreover, brown anoles are territorial and exhibit high site fidelity during the breeding season (Tokarz 1998; Calsbeek 2009), meaning that individuals will likely experience a similar local environment throughout the breeding season. We reasoned that males living in areas characterized by high densities of conspecifics and male-biased OSR would perceive a higher risk of sperm competition and consequently alter their sperm traits to improve their fertilization success. Specifically, we reasoned that the greatest risk of sperm competition would occur in areas of high density and male-biased OSR, while the lowest risk of sperm competition would occur in areas with low density and female-biased OSR. We predicted that males living in high-density areas with a male-biased OSR would produce (1) sperm cells with smaller heads and midpieces, because these phenotypes are associated with increased fertilization success in competitive mating trials in this species (Kahrl and Cox 2015), (2) higher sperm counts, as found in laboratory experiments on other species (Harris and Moore 2004; Ramm and Stockley 2009; Firman et al. 2013; Moatt et al. 2014), and (3) sperm with higher swimming velocity, relative to males living in low-density areas with a female-biased OSR. We tested these predictions by characterizing fine-scale variation in conspecific density and OSR across an entire island population of brown anoles, then asking whether and how variation in each sperm phenotype is related to local density, OSR, and their interaction. Because tradeoffs could constrain males from simultaneously maximizing both quantity and quality of sperm in response to the social environment, we also examined correlations between sperm phenotypes to assess whether such tradeoffs may occur.

## Materials and methods

### Collection of individuals and sperm samples

From May 19 to 28 in 2015, we captured nearly all adult males (*n* = 209) and females (*n *= 465) from a small island population within the Guana Tolomato Matanzas National Estuarine Research Reserve (Palm Coast, Florida, 29°63′N, 81°21′W). Capture probabilities, as estimated with Cormack–Jolly–Seber models using data from prior (April 1–4, 2015) and subsequent (July 28–August 7, 2015) population censuses indicate that we sampled 89% of the males and 86% of the females in the population during this census. We measured snout-vent length (SVL, nearest mm) and body mass (nearest 0.1 g) for each lizard. We kept males in isolation for 24 h prior to collection of a sperm sample, which we obtained by depressing the abdomen and collecting the ejaculate into a microcapillary tube (Kahrl and Cox 2015, 2017).

### Population density and operational sex ratio

**Fig. 1:**
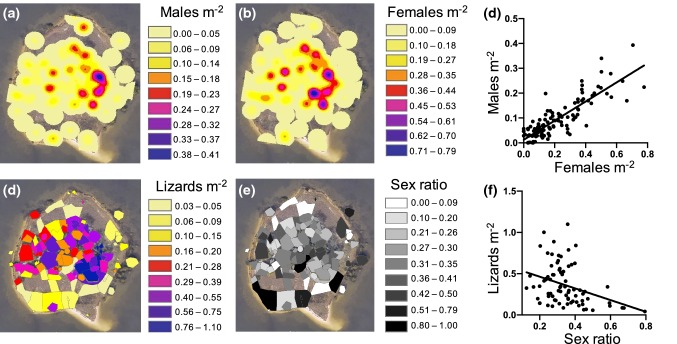
**a**, **b** Heat maps of the island showing estimated gradients in **a** kernel density of males, and **b** kernel density of females using a 5.8-m search radius. Kernel density is an estimate of density for each zone that takes into account the number of lizards found in neighboring zones whose centroids fall within the search radius. **c** Density of males is highly correlated with density of females across the 115 zones to which we mapped lizards; *F*_1,113_ = 302.2425, *P* < 0.0001, *r*^*2*^ = 0.7279. **d**, **e** Map of the island partitioned into zones and showing **d** total density of males and females in each zone (from the quantification of each zone’s centroid in a heat map of total density), and **e** operational sex ratio of each zone (0.0 = only females, 0.5 = equal number of males and females, 1.0 = only males, calculated by dividing male kernel density by total kernel density for each zone). **f** Operational sex ratio and total density are weakly correlated when using the 79 zones that were used in sperm phenotype analyses; *F*_1,77_ = 10.8290, *P* = 0.0015, *r*^*2*^ = 0.1233. Open areas on the maps are zones that were uninhabited by lizards in May 2015. Satellite images of the island are from Google Earth. The color version of this figure is available online

We recorded the location of capture for each lizard by assigning it to the nearest individually numbered tree, shrub, or neighboring area on the island (Fig. S1). After sampling, lizards were returned to their collection location. We recorded GPS measurements of six waypoints on the island by averaging the coordinates taken from those locations over a period of three days at three different times each day. For each numbered tree or shrub, we measured distance and angle bearings from the closest waypoint to the front, back, left, and right edges of the canopy as well as to the trunk. We then constructed a map of the island using ArcGIS (Esri, Redlands, CA) and partitioned the map into zones representing each tree or shrub based on its canopy size, as well as surrounding open areas. The total island area of about 4800 m^2^ was partitioned into 171 zones in this fashion (Fig. S1). We mapped each lizard to the center of the zone corresponding to its location of capture and used the “Kernel Density” tool in ArcGIS to produce a heat map of lizard density across the island when taking into account the density of each zone and of nearby zones whose centroids fell within a search radius (bandwidth) of 5.8 m. This radius was chosen because it represents the active display distance of *Anolis sagrei* (Steinberg et al. 2014), and could, therefore, be considered an approximation of the distance at which a conspecific becomes part of an individual’s local social environment. We used the “Zonal Statistics as Table” tool of ArcGIS to quantify heat maps produced by the “Kernel Density” tool (Fig. 1a, b), then assigned a single density estimate to each zone based on the quantification of the heat map from the zone’s centroid (Fig. 1d). Because the density of males was highly correlated with that of females (Fig. 1c), we used total density for all statistical analyses testing for relationships with density. We calculated the OSR for each zone by dividing male density by total density, such that 0 = only females, 0.5 = even sex ratio, and 1 = only males. All males and females are sexually mature and presumably sexually active in May, so the overall adult sex ratio approximates the OSR.

Our analyses assume that individuals experience relatively stable social environments, such that any social cues that initially trigger plasticity in sperm development are also accurate predictors of the future social environment in which ejaculates will experience postcopulatory selection. This assumption could be violated if lizards frequently move between zones or if the density and OSR of zones shift over time. Therefore, we used data from two census periods (April and July 2015) bracketing the current study (May 2015) to first confirm that (1) lizards tend to remain in or near their zones of capture leading up to the May 2015 period, with 52% of individuals recaptured within the same zone and 66% of individuals recaptured within 5.8 m (one search radius, see above) of their previous zone of capture from April to May 2015, and (2) densities of zones are highly correlated across sampling periods (*r* = 0.8714, *P *< 0.0001, *n *= 96 zones; May–July 2015). However, the OSRs of zones were not correlated across sampling periods (*r* = − 0.0304, *P *= 0.7688, *n* = 96 zones, May–July 2015).

### Sperm traits

To measure the swimming velocity of sperm cells, we suspended ejaculates in Dulbecco modified eagle medium (Gibco, Thermo FischerScientific, Waltham, MA), and immediately added 50 μl of this suspension to a covered well slide. We recorded a 1-min video of each sample at 25 frames per second and 40× magnification using an AmScope digital camera (AmScope, Irvine, CA) with the software ToupView (ToupTek Photonoics, Zhejiang, P.R. China). To measure velocity, we tracked 15 cells per individual for at least 1.6 s (minimum = 40 frames, mean = 54.8 frames) using the Manual Tracking plugin in Image J (NIH, Bethesda, MD). We selected cells by starting in the upper left quadrant of the first frame of the video and tracking every motile cell in that area. We then moved clockwise through each quadrant of the video frame until we had measured the tracks of 15 cells, excluding any that were immobile or visibly impeded by another cell. We calculated the velocity of each cell between each frame of video, then calculated the median curvilinear velocity (VCL) of each cell across all frames (due to the typically right-skewed distribution of VCL values), and finally calculated the median of these median values across 15 cells per male. We used these individual medians as estimates of sperm velocity for subsequent analyses (*n* = 107 males), as distributions were not always normal. We excluded some males from this analysis because their sperm were too crowded on the slide or went out of focus during the recording, preventing accurate measurements of velocity.

From the same sperm sample used for velocity, we fixed the remaining cells from each male in 4% paraformaldehyde, pipetted 10 μl onto a hemocytometer to measure sperm count (*n *= 198 males), then dried the remaining sample onto slides to measure sperm morphology (*n *= 194 males). We stained these slides with Sperm Blue™ (Microptic SL, Barcelona, Spain) and imaged 15 cells per male at 100x magnification with an Olympus Magnafire camera (Olympus America, Melville, NY) using differential interference contrast microscopy. To quantify sperm morphology, we measured the length of the sperm head, midpiece, and flagellum of 15 cells per male (*n *= 194 males) using ImageJ (NIH, Bethesda, MD), then calculated the mean length of the sperm head, midpiece, and flagellum for each male and used these values in our subsequent analyses (Kahrl and Cox 2015, 2017). We chose to measure 15 cells per male because we have previously shown that estimates of both the mean and the coefficient of variation for an individual male’s sperm morphology change relatively little with additional sampling beyond 15 cells (Kahrl and Cox 2015). We have also previously shown that measures of sperm count obtained by our method of collecting ejaculates from males are highly correlated with measures of sperm count obtained by collecting ejaculates from females immediately after mating (Kahrl and Cox 2015).

### Statistical analyses

We performed all statistical tests for relationships between social environment (including both density and OSR) and sperm traits using zones on the island as units of observation because observations from individual lizards are not statistically independent if they occupy the same zones (*n *= 77 zones for sperm morphology and sperm count analyses; *n* = 53 for sperm velocity). For these analyses, we excluded zones with an OSR = 0 (all females, *n *= 11) or 1 (all males, *n* = 3) because these situations do not involve sperm competition. We excluded any males in their second breeding season (*n *= 12) from analyses of sperm count due to a tendency for sperm count to decline with age in this short-lived species (Kahrl, Reedy, Finks, unpublished data). For each zone, we calculated the mean sperm phenotype of all males in that zone. All statistical analyses were performed using JMP (Version 12, SAS institute Inc., Cary, NC).

**Fig. 2:**
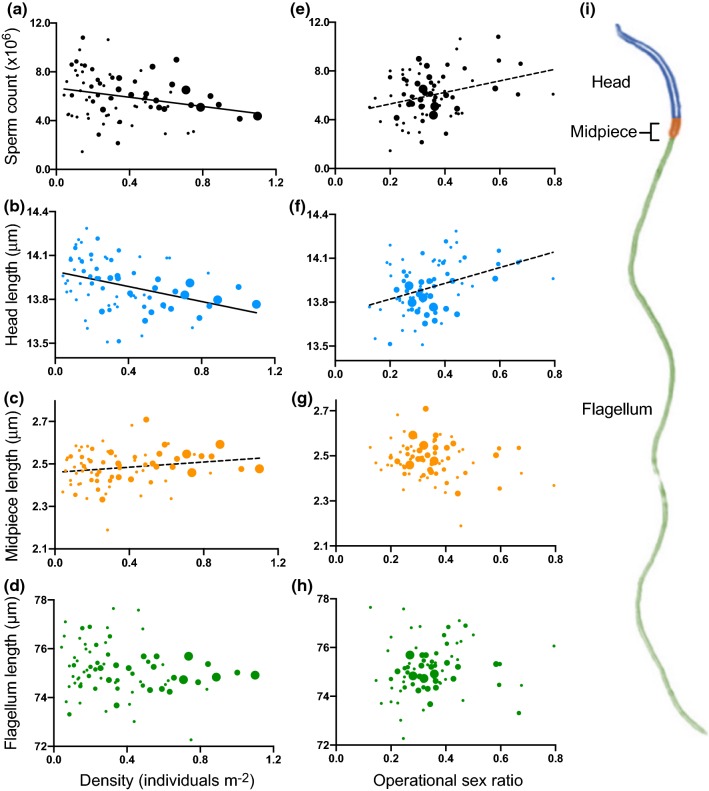
**a**–**d** Scatter plots depicting the relationship between density and **a** sperm count, **b** sperm head length, **c** sperm midpiece length, and **d** sperm flagellum length. **e**–**h** Scatter plots depicting the relationship between operational sex ratio (OSR) and **e** sperm count, **f** sperm head length, **g** sperm midpiece length, and **h** sperm flagellum length. Each point represents one of 77 individual zones in the analysis. Univariate linear regressions are shown for relationships that were significant in both univariate and multivariate analyses (solid line) as well as for relationships that were only significant in univariate analyses (dashed line). Test statistics for univariate and multivariate analyses are reported in Tables 1 and 2, respectively. Each point represents the mean phenotypic measurement of all males occupying that zone, with the size of the symbol corresponding to the number of males contributing to the mean (range 1–8 males). (i) Color-coded *A. sagrei* sperm cell: blue = head, orange = midpiece, green = flagellum. The color version of this figure is available online

To test the hypothesis that sperm traits (sperm morphology, count, and velocity) correlate with differences in local density and OSR, we conducted separate weighted univariate least-squares regressions of each sperm trait on the total density of all lizards (males and females), the density of male lizards, or the OSR. We also analyzed each sperm trait as a response variable in a weighted multivariate model that included total density, OSR, and their interaction as effects. We used total density in our multivariate regressions instead of male density because total density and OSR together should provide a more complete description of the local social environment, whereas male density and OSR should capture much of the same information. Moreover, univariate results from using male density were qualitatively similar to those using total density (Supplemental Table 2). We conducted multivariate analyses (1) to account for the weak negative correlation between density and OSR (Fig. 1f), and (2) because the interaction between density and OSR might be more relevant to sperm competition than the individual effects of either variable (e.g., the relative number of male competitors to potential mates might only matter at high densities). To weight observations, we used the number of males in a zone instead of the inverse of the variance, because some zones contained data from only one male after excluding 2-year-old males or poor sperm samples. We conducted complementary unweighted analyses using individual males (rather than zones) as units of observation, with the density or OSR of the entire zone assigned to each male in the zone. Because non-significant interactions could mask main effects, we also assessed main effects in multivariate regressions conducted the same way but without the interaction between density and OSR. We also examined correlations between all pairwise combinations of ejaculate traits to test for potential tradeoffs.

As a complementary approach to our analyses considering sperm traits separately, we conducted a principal component analysis (PCA) on sperm count, sperm head length, midpiece length, and flagellum length based on individual sperm measurements. Sperm velocity was excluded from this analysis because of a much lower sample size. However, this PCA did not provide useful principal components, as there was no sharp decrease in variance explained between PC1 (37.192%) and PC4 (16.860%; Supplemental Table 1), and we, therefore, performed all analyses considering sperm traits separately, because of insufficient reduction of data dimensionality using PCA.

Body condition has been shown to influence sperm morphology and sperm count in brown anoles (Kahrl and Cox 2015) and could, therefore, confound any correlations between local density and sperm traits, given that density may relate to available food resources. We estimated body condition for each individual by taking the residuals from the regression of log_10_ mass on log_10_ SVL by sex and performed separate univariate least-squares regressions of each sperm trait, density, and OSR on body condition. In the present study, body condition was not associated with sperm count (*F*_1,187_= 0.6095, *P* = 0.4360), head length (*F*_1,191_ = 0.0866, *P* = 0.7689), midpiece length (*F*_1,191_ = 2.3622, *P * = 0.1260), flagellum length (*F*_1,191_ = 3.1408, *P * = 0.0780), or sperm velocity (*F*_1,103_ = 0 .7593, *P* = 0.3856). Body condition was also unrelated to both density (*F*_1,199_ = 0.4861, *P* = 0.4865) and OSR (*F*_1,199_ = 2.6311, *P* = 0.1064). We also tested whether any sperm traits were associated with male body mass and found that body mass was not related to sperm count (*F*_1,187_ =1.5163, *P* = 0.2197), head length (*F*_1,191_ = 0.0355, *P* = 0.8507), midpiece length (*F*_1,191_ = 0.3178, *P * = 0.5736), flagellum length (*F*_1,191_ = 0.0127, *P * = 0.9105), or sperm velocity (*F*_1,103_ = 0.4068, *P* = 0.5250). Therefore, we did not include body condition or body size as covariates in any analysis testing for associations between sperm traits and density or OSR.

## Results

### Spatial distribution of lizards

Lizard density ranged from 0.03 to 1.1 lizards m^−2^ across zones containing at least one lizard, with a mean of 0.30 ± 0.02 lizards m^−2^ (Fig. 1a–d). Male and female densities were highly correlated across zones (Fig. 1c). The OSR ranged from 0.0 to 1.0 across zones (Fig. 1e), with a mean of 0.32 ± 0.02 (female-biased). OSR and total density were weakly correlated, such that areas of higher density tended to have a more female-biased OSR (Fig. 1f).

### Relationships between sperm traits and social environment

Univariate analyses using weighted mean phenotypes for each zone as units of observation revealed that sperm count and head length decreased as density increased, whereas midpiece length increased with density (Table 1; Fig. 2a–c). Flagellum length was not correlated with density (Table 1; Fig. 2d), nor was sperm velocity (Table 1). The results were qualitatively similar when using male density instead of total density (Supplemental Table 2). From our univariate least-squares regressions of each sperm trait on OSR, we found that sperm count and head length increased as zone OSR became more male biased (Table 1; Fig. 2e, f). Midpiece length and flagellum length were not significantly correlated with OSR (Table 1; Fig. 2g, h), nor was sperm velocity (Table 1).

**Table 1:** Results of univariate regressions of density and OSR on sperm traits

Sperm trait	Effect	Zones				Individuals			
		*F*	*P*	*r* ^*2*^	*n*	*F*	*P*	***r*** ^***2***^	***n***
Count	**Density**	**9.4551**	**0.0029**	**0.1120**	77	**8.2369**	**0.0046**	**0.0429**	186
	**OSR**	**7.8170**	**0.0066**	**0.0944**		**6.8961**	**0.0094**	**0.0361**	
Head length	**Density**	**17.2072**	**<** **0.0001**	**0.1866**	77	**12.1902**	**0.0006**	**0.0609**	190
	**OSR**	**9.9179**	**0.0024**	**0.1168**		**7.4483**	**0.0070**	**0.0381**	
Midpiece length	**Density**	**5.2746**	**0.0244**	**0.0657**	77	**4.9544**	**0.0272**	**0.0257**	190
	OSR	2.2557	0.1373	0.0292		2.1699	0.1424	0.0114	
Flagellum length	Density	1.7936	0.1845	0.0234	77	2.0127	0.1576	0.0106	190
	OSR	0.3759	0.5417	0.0050		0.4262	0.5146	0.0023	
Velocity	Density	0.0673	0.7964	0.0013	53	0.0773	0.7816	0.0007	105
	OSR	0.0194	0.8896	0.0004		0.0223	0.8815	0.0002	

Bold values indicate* P* < 0.05

*F*, *P*, and *r*^2^ values are given using both zones and individual males as observations

A multivariate model with density, OSR, and their interaction was a significant predictor of sperm count (by zones: *F*_3,73_ = 4.3025, *P* = 0.0075, *r*^2^ = 0.1503; by individuals: *F*_3,182_ = 3.7014, *P* = 0.0128, *r*^2^ = 0.0575) and sperm head length (by zones: *F*_3,73_ = 8.4608, *P* < 0.0001, *r*^2^ = 0.2580; by individuals: *F*_3,186_ = 5.6988, *P* = 0.0009, *r*^2^ = 0.0842). The full model did not significantly predict sperm midpiece length (by zones: *F*_3,73_ = 2.1166, *P* = 0.1055, *r*^2^ = 0.0800; by individuals: *F*_3,186_ = 2.0014, *P* = 0.1153, *r*^2^ = 0.0313), sperm flagellum length (by zones: *F*_3,73_ = 0.6274, *P* = 0.5996, *r*^2^ = 0.0251; by individuals: *F*_3,186_ = 0.7149, *P* = 0.5442, *r*^2^ = 0.0114), or sperm velocity (by zones: *F*_3,49_ = 0.4325, *P* = 0.7306, *r*^2^ = 0.0258; by individuals: *F*_3,101_ = 0.5017, *P* = 0.6819, *r*^2^ = 0.0147). In regard to specific model effects, as density increased, sperm count and head length decreased (Table 2; Fig. 2a, b). Density was not a significant predictor of midpiece length (Table 2; Fig. 2c), flagellum length (Table 2; Fig. 2d), or sperm velocity (Table 2). OSR was never a significant predictor of any sperm trait (Table 2; Fig. 2e–h). The interaction between density and OSR was not significant in the analyses of any sperm trait (Table 2). Models excluding these non-significant interactions were qualitatively similar to full models with the interaction (Supplemental Table 3; Table 2). All results were also qualitatively similar when using individual males, rather than zones, as units of observations (Tables 1, 2; Fig. S2), so we focus our discussion on the former method using weighted means for each zone.

**Table 2:** Results of multivariate regressions with density, OSR, and their interaction as model effects and sperm traits as response variables

Sperm trait	Model effect	Zones				Individuals			
		*t*	*β*	*P*	*n*	*t*	*β*	*P*	*n*
Count	**Density**	− **2.19**	− **0.4054**	**0.0319**		− **2.03**	− **0.1608**	**0.0439**	
	OSR	1.12	0.2797	0.2660	77	1.04	0.1109	0.2998	186
	Density × OSR	− 0.28	− 0.0645	0.7823		− 0.26	− 0.0256	0.7974	
Head length	**Density**	− **3.53**	− **0.6144**	**0.0007**		− **2.89**	− **0.2225**	**0.0043**	
	OSR	0.23	0.0527	0.8192	77	0.19	0.0191	0.8507	190
	Density × OSR	− 1.82	− 0.3901	0.0734		− 1.49	− 0.1413	0.1377	
Midpiece length	Density	1.96	0.3800	0.0539		1.91	0.1506	0.0583	
	OSR	− 0.02	− 0.0041	0.9873	77	− 0.02	− 0.0016	0.9875	190
	Density × OSR	0.78	0.1875	0.4354		0.76	0.0743	0.4466	
Flagellum length	Density	− 1.10	− 0.2195	0.2752		− 1.17	− 0.0937	0.2421	
	OSR	0.32	0.0849	0.7481	77	0.34	0.0362	0.7312	190
	Density × OSR	0.34	0.0843	0.7330		0.37	0.0360	0.7152	
Velocity	Density	0.02	0.0036	0.9868		0.02	0.0019	0.9858	
	OSR	0.80	0.2356	0.4300	53	0.86	0.1257	0.3934	105
	Density × OSR	1.11	0.3063	0.2730		1.19	0.1634	0.2352	

Bold values indicate* P* < 0.05

*t* ratios, standardized *β* estimates, and *P* values are given using both zones and individual males as observations

### Phenotypic correlations between sperm traits

Correlations between sperm traits were generally weak, but often significant. Among morphological traits, head length and midpiece length were negatively correlated, whereas head length and flagellum length were positively correlated (Table 3). Sperm count was negatively correlated with midpiece length, but it was not correlated with head length or flagellum length (Table 3). Sperm velocity was not correlated with any sperm trait (Table 3).

**Table 3:** Matrix of correlations (*r*) between sperm phenotypes (*P < 0.05)

	Midpiece length	Flagellum length	Sperm count	Sperm velocity
Head length	− 0.3025*	0.1504*	0.0962	0.0170
Midpiece length		− 0.1671*	− 0.2227*	0.1297
Flagellum length			0.0195	0.0311
Sperm count				0.1399

## Discussion

We found that both sperm morphology and sperm count varied with local density in a wild population of *Anolis* lizards, suggesting that males can respond to fine-scale variation in the abundance of potential mates and/or competitors by altering the phenotypes of their ejaculates. Sperm competition theory predicts that males should invest more in ejaculate production as the risk of sperm competition increases (Parker 1993; Parker and Pizzari 2010), however, little is known about whether and how this occurs within wild populations. Although previous studies have experimentally demonstrated effects of the social environment on ejaculate phenotypes in captive males (Crean and Marshall 2008; Ramm and Stockley 2009; Immler et al. 2010; Kelly and Jennions 2011; Firman et al. 2013; Moatt et al. 2014; Giannakara et al. 2016) or the effects of interpopulation differences in risk of sperm competition on ejaculate phenotypes (Dziminski et al. 2010; Álvarez et al. 2013), ours is the first study to provide evidence that sperm traits respond to fine-scale natural variation in the social environment within a single wild population. Below, we discuss how the spatial patterns in sperm morphology and sperm count that we observed may reflect adaptive plasticity in male reproductive phenotypes.

We found that length of the sperm midpiece increased, whereas length of the sperm head decreased, as local density increased. This relationship between density and sperm head length was robust when accounting for variation in OSR. The only significant predictor of midpiece length was the fully reduced univariate density analysis. In competitive fertilization trials on captive brown anoles, smaller sperm heads were associated with increased paternity (Kahrl and Cox 2015). Therefore, the negative correlation that we observed between head length and population density may indicate that individuals at higher densities produced competitively superior sperm. Longer sperm heads can increase drag and lower velocity (Humphries et al. 2008; Lüpold et al. 2009), though we found no association between head length and velocity in this study. Larger midpieces are often viewed as adaptive because they contain more mitochondria and are associated with increased sperm performance (e.g., velocity and ATP concentration) and male fitness in many species (Vladić et al. 2002; Lüpold et al. 2009; Firman and Simmons 2010; Fisher et al. 2016). Although we did not find a positive correlation between midpiece length and sperm velocity in this study, larger midepieces could promote cell longevity (Smith and Ryan 2010), which could be more important than velocity in species with internal fertilization (Smith 2012). Moreover, experimental increases in the perceived risk of reproductive competition resulted in the production of sperm with larger midpieces in Gouldian finches (*Erythrura gouldiae*; Immler et al. 2010). However, without data linking sperm traits to reproductive success in our study, it is difficult to assess whether the correlations we observed are adaptive. Although larger midpieces are often viewed as adaptive, we predicted that we would see smaller midpieces in more competitive environments because smaller midpieces were associated with increased paternity in a previous study of brown anoles (Kahrl and Cox 2015). Thus, the positive correlation between density and midpiece size that we observed may simply reflect the negative phenotypic correlation that we observed between sperm head and midpiece length (Table 3).

We also found that sperm count decreased with local density in our wild population. Interestingly, this result is contrary to the general prediction that males should increase sperm production in response to high levels of sperm competition (Parker 1993), which is supported by several experiments in which the risk of sperm competition was altered (Ramm and Stockley 2009; Kelly and Jennions 2011; Firman et al. 2013; Moatt et al. 2014). The negative correlation between density and sperm count that we observed could result from different allocation strategies that are dependent on density. For example, we found a negative correlation between sperm count and midpiece length, which suggests a possible tradeoff between sperm quality and quantity (Parker et al. 1996; Immler et al. 2011). The negative correlation between sperm count and density that we observed may not represent a tradeoff between quantity and quality of sperm, but could simply arise because males favor mate guarding over sperm production at high densities (Alonzo and Warner 2000). Alternatively, males at high densities may mate more frequently and, therefore, have fewer sperm available at any particular time. This phenomenon has been observed in mosquito fish (*Gambusia holbrooki*), where males raised in environments with a high risk of sperm competition mated more frequently and had fewer sperm remaining at the end of the experiment, relative to males raised in environments with low risk of sperm competition (Evans et al. 2003).

Whereas density was correlated with several sperm traits, we found no relationship between OSR and sperm traits after accounting for density. This lack of association between sperm phenotypes and OSR may be because the island sex ratio is heavily female-biased (mean OSR = 0.32, approximately two females per male). Although individual zones that we included in our analysis ranged from 0.1 to 0.8 in OSR, only 8 of the 77 zones had male-biased sex ratios (i.e., OSR > 0.5), and only 10.5% of the males in the population as a whole, occupied zones with male-biased OSR. The lack of stability of the OSR between May and July 2015 may indicate that our measurement of OSR is subject to error. Because we lack data on female receptivity, our estimation of OSR assumes that all adult females are reproductive and receptive. This estimation may differ from the more dynamic and actual OSR of receptive females to sexually active males (Kvarnemo and Ahnesjo 1996), which males may have used as a cue during sperm production. Alternatively, there may be no association because males may not respond to OSR if it is an unstable cue.

Our findings suggest that sperm morphology and sperm count respond more strongly to the local density of potential mates and competitors than to the operational sex ratio, and our study is the first to show such a relationship within a wild population. Our study broadens the implications of sperm competition theory by suggesting that it can be extended to fine-scale natural variation in the social environment found within wild populations.

## Electronic supplementary material

Below is the link to the electronic supplementary material.

Supplementary material 1 (DOCX 2391 kb)

## Data Availability

Data available from the Dryad Digital Repository: 10.7291/D1308K.
