# Supplementary material for: Sperm morphology and count vary with fine-scale changes in local density in a wild lizard population
Source: Oecologia. 2019 Oct 17;191(3):555–64. doi: 10.1007/s00442-019-04511-z (PMC6825022; doi:10.1007/s00442-019-04511-z)
Supplement: Supplementary file 1 — Supplementary material 1 (DOCX 2391 kb) [file 442_2019_4511_MOESM1_ESM.docx]

**Supplemental Table 1.** Loading matrix of Principal Components Analysis for four sperm traits. Percentage of variation explained by each principle component is given in parentheses.

|  | **PC1** (37.192) | **PC2** (24.692) | **PC3** (21.256) | **PC4** (16.860) |
| --- | --- | --- | --- | --- |
| **Head Length** | 0.67647 | 0.16334 | -0.56903 | 0.43808 |
| **Midpiece Length** | -0.76385 | 0.11062 | 0.11526 | 0.62531 |
| **Flagellum Length** | 0.42731 | 0.72342 | 0.53767 | 0.07064 |
| **Count** | 0.51380 | -0.65224 | 0.47339 | 0.29411 |

| **Sperm Trait** | **Effect** | **Zones** | | | |  | **Individuals** | | | |
| --- | --- | --- | --- | --- | --- | --- | --- | --- | --- | --- |
|  |  | ***F*** | ***P*** | ***r^2^*** | ***n*** | ***F*** | | ***P*** | ***r^2^*** | ***n*** |
| Count | **Total** | **9.4551** | **0.0029** | **0.1120** | 77 | **8.2369** | | **0.0046** | **0.0429** | 186 |
|  | **Male** | **5.5470** | **0.0211** | **0.0689** |  | **4.9808** | | **0.0268** | **0.0264** |  |
| Head Length | **Total** | **17.207**2 | **<0.0001** | **0.1866** | 77 | **12.1902** | | **0.0006** | **0.0609** | 190 |
|  | **Male** | **13.2858** | **0.0005** | **0.1505** |  | **9.7077** | | **0.0021** | **0.0491** |  |
| Midpiece Length | **Total** | **5.2745** | **0.0244** | **0.0657** | 77 | **4.9544** | | **0.0272** | **0.0257** | 190 |
|  | **Male** | **4.3776** | **0.0398** | **0.0552** |  | **4.1406** | | **0.0433** | **0.0216** |  |
| Flagellum Length | Total | 1.7936 | 0.1845 | 0.0234 | 77 | 2.0127 | | 0.1576 | 0.0106 | 190 |
|  | Male | 1.1994 | 0.2769 | 0.0157 |  | 1.3517 | | 0.2465 | 0.0071 |  |
| Velocity | Total | 0.0673 | 0.7964 | 0.0013 | 53 | 0.0773 | | 0.7816 | 0.0007 | 105 |
|  | Male | 0.0167 | 0.8976 | 0.0003 |  | 0.0192 | | 0.8900 | 0.0002 |  |

**Supplemental Table 2.** Comparison of univariate analyses testing for effects of total (male and female) density or male density on sperm traits, with significant effects in bold. Statistics are reported using both zones and individual males as units of observation.

| **Sperm Trait** | **Effect** | **Zones** | | | | **Individuals** | | | |
| --- | --- | --- | --- | --- | --- | --- | --- | --- | --- |
|  |  | ***t*** | ***ß*** | ***P*** | ***n*** | ***t*** | ***ß*** | ***P*** | ***n*** |
| Count | **Density** | **-2.19** | **-0.3963** | **0.0319** | 77 | **-2.02** | **-0.1571** | **0.0448** | 186 |
|  | OSR | 1.80 | 0.3269 | 0.0753 |  | 1.67 | 0.1296 | 0.0973 |  |
| Head Length | **Density** | **-3.21** | **-0.5578** | **0.0020** | 77 | **-2.66** | **-0.2020** | **0.0084** | 190 |
|  | OSR | 1.90 | 0.3307 | 0.0613 |  | 1.58 | 0.1198 | 0.1162 |  |
| Midpiece Length | Density | 1.85 | 0.3528 | 0.0678 | 77 | 1.80 | 0.1399 | 0.0735 | 190 |
|  | OSR | -0.72 | -0.1377 | 0.4716 |  | -0.70 | -0.0546 | 0.4832 |  |
| Flagellum Length | Density | -1.19 | -0.2317 | 0.2391 | 77 | -1.26 | -0.0990 | 0.2085 | 190 |
|  | OSR | 0.13 | 0.0248 | 0.8991 |  | 0.14 | 0.0106 | 0.8925 |  |
| Velocity | Density | -0.22 | -0.0477 | 0.8258 | 53 | -0.24 | -0.0254 | 0.8121 | 105 |
|  | OSR | 0.05 | 0.0098 | 0.9637 |  | 0.05 | 0.0053 | 0.9608 |  |

**Supplemental Table 3.** Results of multivariate regressions with sperm traits as response variables and density and OSR as main effects without interactions. Significant model effects are in bold. Overall model was a significant predictor of sperm count (by zones: *F*_2,74_ = 6.4964, *P* = 0.0025, *r*^2^ = 0.1494; by individuals: *F*_2,183_ = 5.5474, *P* = 0.0046, *r*^2^ = 0.0572) and sperm head length (by zones: *F*_2,74_ = 10.7081, *P* = <0.0001, *r*^2^ = 0.2245; by individuals: *F*_2,187_ = 7.3889, *P* = 0.0008, *r*^2^ = 0.0732). Overall model was not a significant predictor of sperm midpiece length (By zones: *F*_2,74_ = 2.8823, *P* = 0.0623, *r*^2^ = 0.0723; by individuals: *F*_2,187_ = 2.7174, *P* = 0.0687, *r*^2^ = 0.0282), sperm flagellum length (by zones: *F*_2,74_ = 0.8931, *P* = 0.4137, *r*^2^ = 0.0236; by individuals: *F*_2,187_ = 1.0103, *P* = 0.3661, *r*^2^ = 0.0107), and sperm velocity (by zones: *F*_2,50_ = 0.0340, *P* = 0.9666, *r*^2^ = 0.001**4**; by individuals: *F*_2,102_ = 0.0395, *P* = 0.9613, *r*^2^ = 0.0001).

**Supplemental Figure 1**. Satellite image of the study island from Google earth mapped into zones using ArcGIS. Unique ID codes for each zone (e.g., H72F) refer to the study island (H), the number of the tree at the center of the zone (1-100), or the area of small shrubs and vegetation (M/Y) or open grass, mud, and sand (F) adjacent to a particular tree. Zone IDs with an underscore character indicate areas located between two numbered trees (e.g., H29_H31).

**Supplemental Figure 2**. (a-d) Scatter plots depicting the relationship between density and (a) sperm count (*n* = 186), (b) sperm head length (*n* = 190), (c) sperm midpiece length (*n* = 190), and (d) sperm flagellum length (*n* = 190). Scatter plots depicting the relationship between operational sex ratio (OSR) and (e) sperm count (*n* = 186), (f) sperm head length (*n* = 190), (g) sperm midpiece length (*n* = 190), and (h) sperm flagellum length (*n* = 190). Univariate linear regressions are shown for relationships that were significant (*P* < 0.05) in both univariate and multivariate analyses (solid line) as well as for relationships that were only significant in univariate analyses (dashed line). Test statistics for univariate and multivariate analyses are reported in the main text tables 1 and 2, respectively. (i) Color-coded *A. sagrei* sperm cell: blue = head, orange = midpiece, green = flagellum.


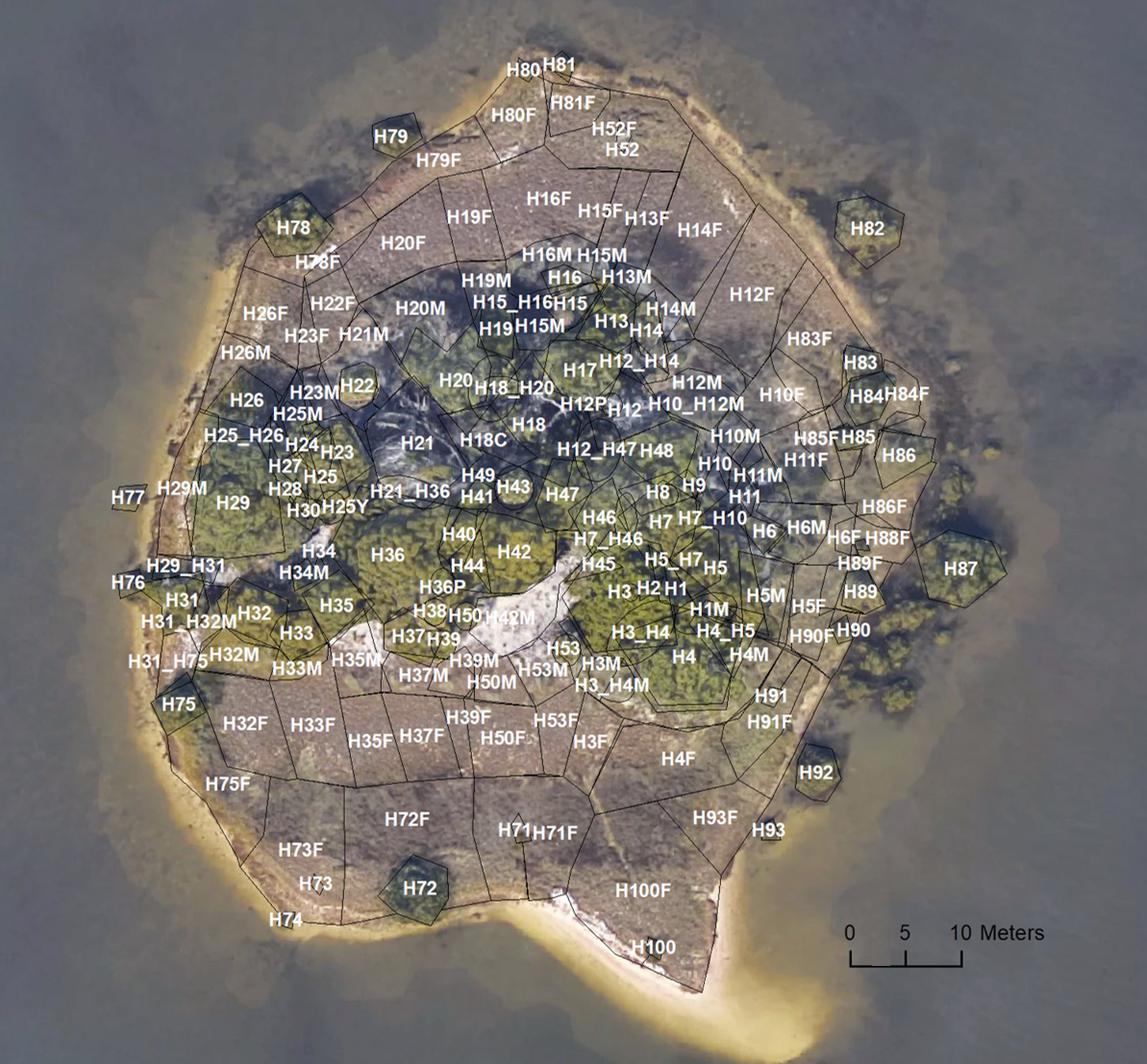


**Supplemental Figure 1.**

**Supplemental Figure 2**.
